# The estrous cycle moderates the food and body weight suppressive effects of glucagon‐like peptide‐1 receptor agonism

**DOI:** 10.1111/dom.70177

**Published:** 2025-09-29

**Authors:** Sarah V. Applebey, Allison G. Xiao, Benjamin C. Reiner, Matthew R. Hayes

**Affiliations:** ^1^ Department of Psychiatry, Perelman School of Medicine University of Pennsylvania Philadelphia Pennsylvania USA

## Abstract

**Aims:**

Emerging data suggest more young women than men are prescribed weight loss pharmacotherapies targeting the glucagon‐like peptide‐1 receptor (GLP‐1R). However, preclinical literature has largely used male animals to characterize the neural mechanisms underlying the weight loss abilities of GLP‐1R agonists (GLP‐1RAs), highlighting a need for female‐specific investigations. Recently, we described data pointing to the female estrous cycle as a possible moderator of GLP‐1RA's effects in rats. Expression of brainstem *Glp1r* and the GLP‐1 precursor gene, *Gcg*, increased during two estrous phases, proestrus and estrus (P/E), compared to males and compared to other phases, metestrus and diestrus (M/D). On this basis, we hypothesized that the weight‐reducing effects of GLP‐1RAs may be potentiated during P/E.

**Materials and Methods:**

In separate experiments, we determined whether timing administration of acute liraglutide or chronic semaglutide to either P/E or M/D would moderate food intake and weight loss in female rats maintained on a high fat diet. We also used qPCR to explore estrous cycle‐dependent variation in *Glp1r* within widely distributed nuclei relevant to energy balance control.

**Results:**

GLP‐1RA administration during P/E, compared to M/D, enhanced the intake‐suppressive effects of liraglutide and semaglutide. Moreover, semaglutide administered only during P/E led to greater body weight loss compared to M/D‐administered semaglutide. We also observed greater *Glp1r* expression in P/E compared to M/D in multiple nuclei.

**Conclusions:**

GLP‐1RAs administered in P/E lead to significantly greater body weight loss via reduction in food intake. Collectively, these data may have translational implications for the timing of GLP‐1RA administration across the menstrual cycle.

## 1 INTRODUCTION

Glucagon‐like peptide‐1 receptor (GLP‐1R) agonists (GLP‐1RAs) are highly effective for treating type 2 diabetes and obesity. Having gained substantial traction for their weight loss effects, young women now largely drive the demand for FDA‐approved GLP‐1RAs,^1^ and clinical literature points to additional female‐specific mechanisms in responsivity to GLP‐1RAs. Compared to men, women show a greater percentage of body weight loss in response to GLP‐1RAs, including FDA‐approved exenatide,^2^, ^3^ liraglutide,^4^ semaglutide,^5^, ^6^ and dulaglutide.^7^, ^8^ Historically, however, most preclinical studies investigating GLP‐1R agonists used only male animal models^9^, ^10^, ^11^ to avoid confounding anorexigenic effects associated with the ovarian cycle.^12^ Nonetheless, a growing body of data point to female‐specific neural underpinnings of GLP‐1(RAs)^11^, ^13^ and indicate female rats can demonstrate heightened sensitivity to acute GLP‐1RA compared to males.^14^ However, the impact of the ovarian cycle itself on the therapeutic efficacy of GLP‐1RAs is unclear.

In both sexes, GLP‐1RAs promote weight loss by suppressing caloric intake by activating multiple GLP‐1R‐expressing neural populations in the central nervous system (CNS).^15^, ^16^ Principal among CNS sites of action, GLP‐1R populations in the nucleus tractus solitarius (NTS), a hindbrain nucleus essential to energy balance control,^17^ are critical for the anorectic effects of endogenous GLP‐1 and peripherally administered GLP‐1RAs.^9^, ^18^, ^19^, ^20^ The caudal NTS is also the predominant source of brain preproglucagon neurons that synthesize endogenous GLP‐1 and project to many GLP‐1R‐expressing nuclei of relevance to energy balance control.^21^, ^22^ Targeted activation of GLP‐1Rs in many of these nuclei leads to the suppression of food intake or food motivation, such as the nucleus accumbens (NAc),^23^ bed nucleus of the stria terminalis (BNST),^24^ hypothalamic paraventricular nucleus (PVN),^25^ thalamic paraventricular nucleus (PVT),^26^ central amygdala (CeA),^27^ arcuate nucleus of the hypothalamus (Arc),^28^ and the ventral tegmental area (VTA).^29^, ^30^ For several of these nuclei, there is some evidence for sex‐divergent behaviours following GLP‐1R activation.^11^

It is well established that the ovarian hormone estradiol is responsible for the cyclic suppression of feeding within the ovarian cycle, termed the estrous cycle in rodents or menstrual cycle in women. In rodents, circulating estradiol levels peak during proestrus (P), one of four estrous phases, to promote ovulation. Estradiol levels return to baseline by the subsequent phase, estrus (E),^31^ but have delayed effects that reduce appetitive and consummatory ingestive behaviour typically in E^12^, ^32^ through transcriptional regulation of estradiol‐responsive genes.^31^ The ability of the estrous cycle and estradiol to suppress food intake is best characterized by work on the satiation signal cholecystokinin (CCK). Endogenous CCK is not only necessary and sufficient for the cyclic suppression of feeding in E,^33^ but exogenous estradiol similarly enhances the satiating potency^34^ and neural processing of CCK.^35^ While a few studies have investigated the interaction between GLP‐1R action and the ovarian cycle,^11^ multiple studies demonstrate exogenous estradiol augments the anorexigenic or anti‐dipsogenic effects of GLP‐1RAs.^36^, ^37^, ^38^, ^39^ Moreover, we recently observed increased brainstem expression of the GLP‐1 precursor gene, glucagon (*Gcg*) in both P and E, while *Glp1r* expression was greater during E.^40^ This increase had functional significance, as brainstem GLP‐1R antagonism restored expected overconsumption only during combined P and E (P/E) compared to the other two phases, combined metestrus (M) and diestrus (D) (M/D). Similarly, whole hypothalamic *Glp1r* is increased during late P^41^ and may underscore the ability of lateral hypothalamic GLP‐1R activation to attenuate food reinforcement in E, but not M/D.^42^

Considering these data, we hypothesized that females may demonstrate increased sensitivity to GLP‐1RAs in P/E. Greater endogenous brain GLP‐1 or GLP‐1R expression during P/E may potentiate the effects of GLP‐1RAs. Here, we explore this hypothesis by investigating the ability of the rat estrous cycle to moderate the food intake and body weight reducing effects of two U.S. FDA‐approved GLP‐1RAs, liraglutide and semaglutide, and by characterizing estrous cycle‐dependent variation in *Glp1r* expression in distributed nuclei. Our findings may have therapeutic translational implications for the timing of GLP‐1R administration across the menstrual cycle.

## 2 MATERIALS AND METHODS

### 2.1 Animals

Adult female Sprague–Dawley rats (Charles River) were housed individually in hanging metal wire cages at the University of Pennsylvania, under a 12:12‐h dark:light cycle in a temperature‐ and humidity‐controlled vivarium. All animals were provided ad libitum access to water and either lab chow diet (Purina LabDiet) or high‐fat diet (60% kcal fat, Research Diets #D12492) when specified. The University of Pennsylvania Institutional Animal Care and Use Committee approved all protocols (803503).

### 2.2 Estrous cycle tracking/vaginal cytology

We monitored the female estrous cycle using daily vaginal lavage according to established protocols^43^ and as previously described.^40^ We defined each phase as the 24 h starting with dark cycle onset. For lean animals, we collected samples near the end of each phase, 8 h into the light cycle based on.^44^ As HFD‐maintained females often demonstrate greater variability in estrous phase length,^45^ we adjusted our collection time to be closer to optimal (dark‐to‐light transition)^46^ for HFD‐maintained females and collected samples 4–6 h before light onset.

### 2.3 Drug preparation

Liraglutide and semaglutide were obtained from Cayman Chemical. We dissolved 50 μg/kg of liraglutide in 0.9% saline and dissolved 20 nanomol/kg of semaglutide in its vehicle [40 mM tris HCl buffer and 0.01% Tween 20 (pH 8.0)] based on previous effects on food intake.^47^, ^48^ All injections were administered intraperitoneally directly before dark cycle onset.

### 2.4 Liraglutide administration

To model conditions in which GLP‐1RAs are administered in humans, we maintained female rats (*n* = 30) on a high fat diet (HFD) for 7 weeks. Upon arrival, animals averaged 266 ± 1 g and were 367 ± 5 g at the experimentation start. We habituated all animals to handling and intraperitoneal injections prior to testing in the home cage. Pica, the consumption of nonnutritive kaolin clay, is a well‐documented behaviour indicative of the nausea/malaise accompanying GLP‐1RAs.^49^ To measure pica, we habituated animals to ad libitum access to kaolin pellets (K50001, Research Diets) for at least 5 days prior to testing.

In a counterbalanced, within‐subjects design, we administered liraglutide to animals in P/E (once during either P or E) or M/D (once during either M or D). These phases are frequently pooled due to high oestrogen levels (P) or effects (E) or low oestrogen levels/influence (M and D),^50^, ^51^ thus we combined animals in M/D and P/E for all experiments. We attempted to keep the injection phase consistent for each animal so an animal in E during the first P/E injection would continue to be injected in E, not P. We measured food intake 1, 3, 6, and 24 h post‐injection and determined changes in body weight and kaolin intake after 24 h. Each treatment was separated by at least 72 h. *n* = 3 animals were excluded due to inconsistent estrous cycles.

### 2.5 Semaglutide administration

After an ~2 week washout, we examined the impact of chronically administering a longer‐acting synthetic GLP‐1RA, semaglutide, in the same HFD‐maintained female cohort (*n* = 30). Animals weighed 383 ± 6 g at experiment start. We balanced rats into three treatment groups based on body weight and estrous cycle length (4–5 days). All groups received one injection during P/E and one during M/D, with the injection identity (vehicle or semaglutide) varying by group. One group received vehicle injections only (*n* = 8), one group received a semaglutide injection during M/D and a vehicle injection during P/E (*n* = 10), and the third received semaglutide during P/E and vehicle during M/D (*n* = 11). We attempted to keep injection phases consistent for each animal. At least 48 h elapsed between each injection, and the semaglutide injection occurred once per cycle, separated by at least 96 h. See Figure S1 for example timelines and further detail. We administered treatment for 24 days, recording food and body weight daily. For each semaglutide‐treated group, *n* = 2 had inconsistent estrous cycle length and received 5 instead of 6 semaglutide injections. As animals received injections on different dates, to compare time points, we analysed food intake every 4 days based on a 4‐day estrous cycle. To determine percent change in body weight, we subtracted a 4‐day body weight average from starting weight. Given the variation in each animal's estrous cycle, we could not fully anticipate each phase, and some animals received semaglutide injections during phases that did not align with their assigned group. We excluded one animal injected during the incorrect phase for ≥3/6 of injections.

### 2.6 Tissue extraction for quantitative polymerase chain reaction (qPCR)

To investigate the impact of estrous cycle phase on *Glp1r* expression in lean animals, we utilized cortex tissue from the chow‐maintained female animals from which we previously collected NTS/AP tissue.^40^ To examine the effect of estrous cycle phase on *Glp1r* and *Gcg* expression in HFD‐maintained animals, we used the same cohort from the aforementioned liraglutide and semaglutide experiments. Vehicle‐treated animals recovered for 10–11 days before brain extraction. Semaglutide‐treated animals recovered for a minimum of 4 weeks to weight‐match the vehicle group (with a maximum of 5 additional days depending on the day of each animal's final semaglutide injection and counterbalanced sacrifice date). See Supporting Information Appendix for specific details (Figure S2) and brain extraction and qPCR methodology. Given the time and experimental differences between lean and HFD‐maintained animals, we did not compare these groups.

### 2.7 Statistical analyses

We used GraphPad Prism 10.4.1 (GraphPad Software) to analyse data. We analysed behavioural data using paired t‐tests and repeated‐measures or mixed ANOVAs followed by.

Holm‐Šídák's post‐hoc tests. Gene expression data were analysed using paired t‐tests. Specific tests and results, including main effects and interactions, are presented in Table S2. We considered *p* < 0.05 significant and denote the significance of relevant post‐hocs in figure legends. All data are expressed as mean ± SEM.

## 3 RESULTS

### 3.1 *Glp1r* expression across the estrous cycle in lean animals

**FIGURE 1:**
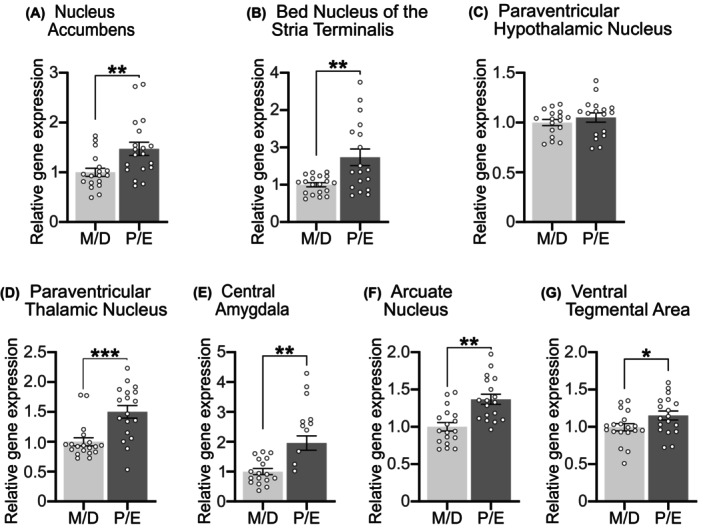
In lean female rats, *Glp1r* expression was greater during combined Proestrus/Estrus (P/E) compared to animals in Metestrus/Diestrus (M/D) in the (A) Nucleus Accumbens, *p* = 0.0055, (B) Bed Nucleus of the Stria Terminalis, *p* = 0.0023, (D) Paraventricular Thalamic Nucleus, *p* = 0.0003, (E) Central Amygdala, *p* = 0.0016, (F) Arcuate Nucleus, *p* = 0.0002, and (G) Ventral Tegmental Area, *p* = 0.045, but not in the (C) Paraventricular Hypothalamic Nucleus, *p* = 0.378. *n* = 17–20/group, between‐subjects design. *p < 0.05; **p < 0.01; ***p < 0.001.

Previously, we showed *Glp1r* and *Gcg* expression in the NTS/AP is increased in P/E compared to M/D.^40^ Using archived fresh frozen brains from these same lean female rats, we determined whether the estrous cycle impacts *Glp1r* expression in select neural nuclei (Figure 1). We selected each region based on literature showing that GLP‐1Rs activation in those nuclei decreased ingestive behaviour, including the NAc,^30^ BNST,^24^ PVN,^25^ PVT,^26^ CeA,^27^ Arc,^28^ and VTA.^29^, ^30^ Compared to expression levels in M/D, we found increased *Glp1r* expression in animals in P/E in the NAc (Figure 1A) and BNST (Figure 1B) (both *p* < 0.005), but not the PVN (Figure 1C). *Glp1r* expression during P/E also increased in the PVT (Figure 1D), CeA (Figure 1E), Arc (Figure 1F), and VTA (Figure 1G) (all *p* < 0.05). Given these estrous cycle‐dependent changes, we next investigated whether estrous phases associated with increased G*lp1r* would enhance the anorectic effects of two GLP‐1RAs.

### 3.2 Liraglutide administration

**FIGURE 2:**
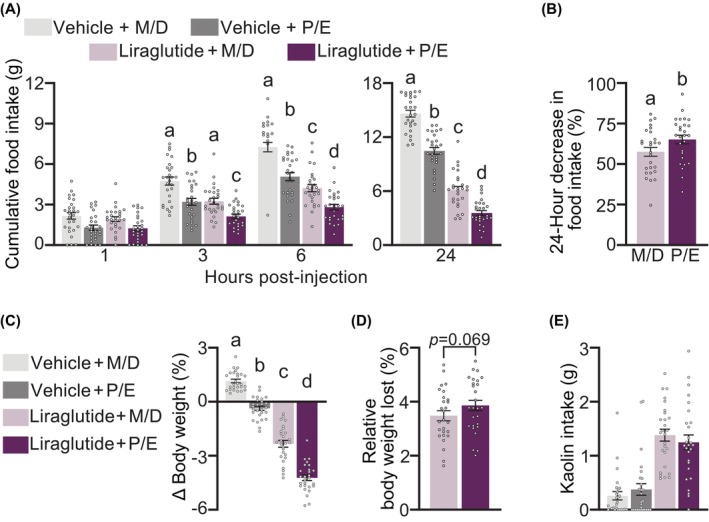
Effect of estrous cycle phase on the efficacy of acute liraglutide (50 μg/kg; intraperitoneal) in female rats maintained on high‐fat diet. In a within‐subjects design, animals were exposed to vehicle or liraglutide while in Proestrus/Estrus (P/E) or Metestrus/Diestrus (M/D). (A) Cumulative food intake in grams (g) at 1, 3, and 6 h (left) and 24 h (right) post‐injection. Administration during P/E potentiated liraglutide's intake‐suppressive effects compared to injections in M/D at 24 h. (B) Percent suppression of cumulative food intake 24 h post‐injection. Relative to their respective vehicle injections, liraglutide administration in P/E produced a greater percent suppression of cumulative food intake compared to M/D. (C) Percentage of body weight change 24 h after liraglutide administration. There were individual main effects of estrous cycle phase and liraglutide to reduce body weight. (D) Percentage of weight lost 24 h post‐injection relative to vehicle injection when animals were in M/D or P/E. P/E injection increased the intake‐suppressive effect of liraglutide compared to M/D. Liraglutide provoked a similar percentage of weight lost in P/E and M/D. (E) Kaolin intake in grams. There was a main effect of liraglutide administration to increase kaolin intake. There was no main effect of estrous cycle phase nor interaction. Within each time point, means with different letters are significantly different from each other (*p* < 0.01). *n* = 27/group, within‐subjects design.

We first investigated whether estrous phase would moderate the anorectic, weight‐reducing effects of acutely delivered liraglutide (50 μg/kg) in HFD‐maintained female rats (Figure 2). Liraglutide had a main effect, significantly reducing cumulative food intake after 3, 6, and 24 h by 1.29 ± 0.24, 2.65 ± 0.32, and 7.68 ± 0.40 g respectively. There was also an effect of estrous cycle phase, with rats in P/E consuming less than animals in M/D at all time points (1 h: 0.78 ± 0.20 g and by 24 h: 3.36 ± 0.35 g less). At 24 h, there was a significant interaction between estrous phase and liraglutide (Figure 2A). Liraglutide‐treated animals in P/E consumed less (*p* < 0.0001) than vehicle‐treated animals in P/E (by 6.91 ± 0.34 g) and liraglutide‐treated animals in M/D (by 2.56 ± 0.34 g). Moreover, at 24 h, animals in P/E showed a significantly greater percent suppression of cumulative food intake (P/E: 65.1% vs. 57.57% in M/D) from their respective vehicle treatments (Figure 2B). Conversely, liraglutide and estrous cycle had individual main effects on body weight (both *p* < 0.0001) with no interaction (Figure 2C). Liraglutide‐injected animals lost a greater (3.67 ± 0.16%) body weight percentage compared to vehicle‐injected animals, and animals in P/E lost more body weight (1.70 ± 0.11%) than animals in M/D. These separate additive effects resulted in liraglutide‐treated animals in P/E losing the greatest percentage of weight after injection. However, relative to their respective vehicle treatments, liraglutide‐treated animals in both M/D and P/E showed a similar magnitude of weight loss (M/D: 3.48% vs. P/E: 3.86%; *p* = 0.069) (Figure 2D). Finally, liraglutide treatment, but not estrous cycle, triggered a pica response (i.e., kaolin intake) (Figure 2E). Liraglutide administration increased 24‐h kaolin intake compared to vehicle‐treated animals by 1.00 ± 0.11 g (*p* < 0.0001).

### 3.3 Semaglutide administration

**FIGURE 3:**
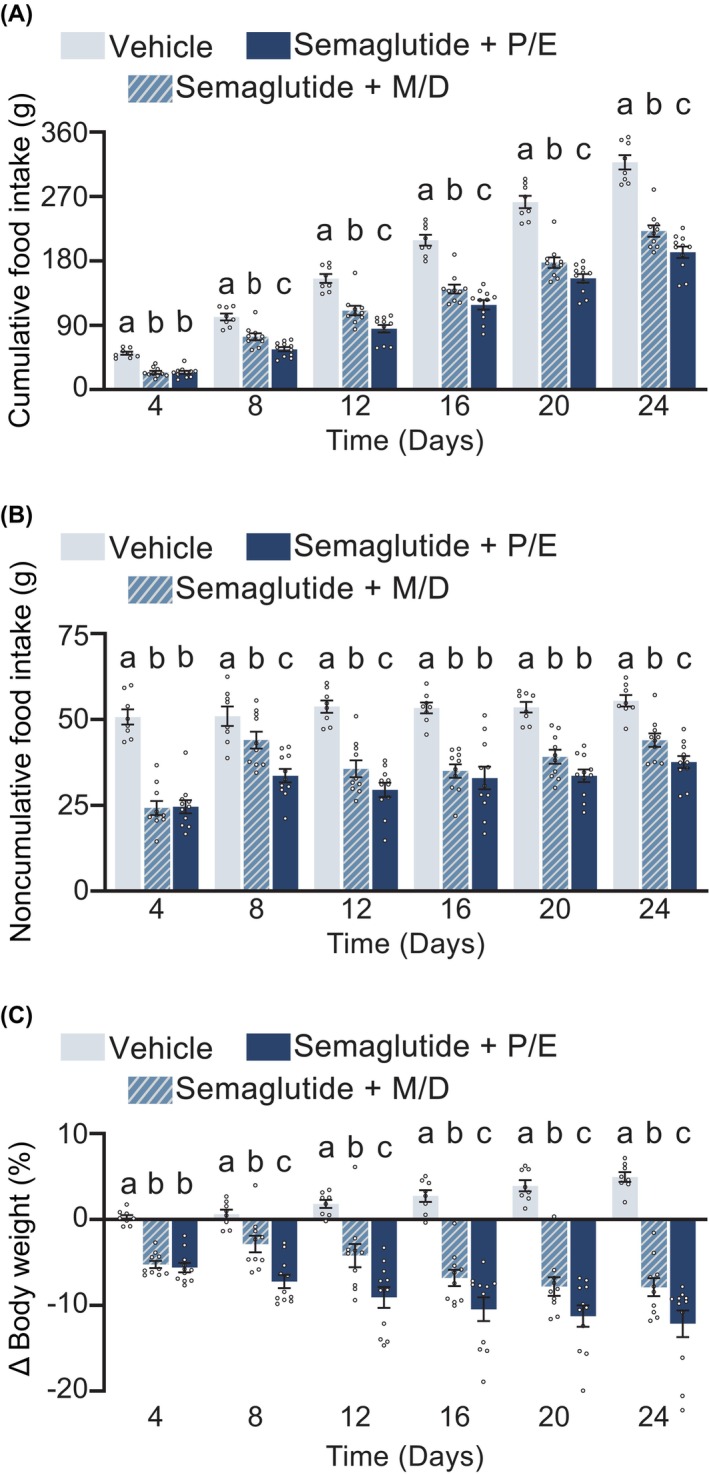
Effect of estrous cycle phase on the efficacy of chronic semaglutide (20 nmol/kg; intraperitoneal) in female rats maintained on high‐fat diet. (A) Cumulative food intake in grams (G). There was a main effect of condition. At 8, 12, 16, 20, and 24 days from first injection, injections during Proestrus/Estrus (P/E) potentiated the intake‐suppressive effects of semaglutide, with rats consuming less than animals injected during Metestrus/Diestrus (M/D) (*p* = 0.0042, *p* = 0.0048, *p* = 0.0240, *p* = 0.0339, *p* = 0.0166, respectively). (B) Noncumulative food intake in grams (g). There was a main effect of condition, time, and interaction. At time bins ending at day 8, 12, and 24 days from first injection, injections during (P/E) potentiated the intake‐suppressive effects of semaglutide, with rats consuming less than animals injected during Metestrus/Diestrus (M/D) (*p* = 0.0011, *p* = 0.0400, *p* = 0.038, *p* = 0.0339, *p* = 0.0166, respectively). (C) Percentage of body weight change from baseline. There was a main effect of group, time, and an interaction. At 8, 12, 16, 20, and 24 days from first injection, injections during P/E increased the weight‐reducing effects of semaglutide, compared to animals injected during M/D (*p* = 0.0038, *p* = 0.0006, *p* = 0.009, *p* = 0.0139, *p* = 0.0020, respectively). Within each time point, means with different letters are significantly different from each other (*p* < 0.05). *n* = 8–11/group, between‐subjects design.

**FIGURE 4:**
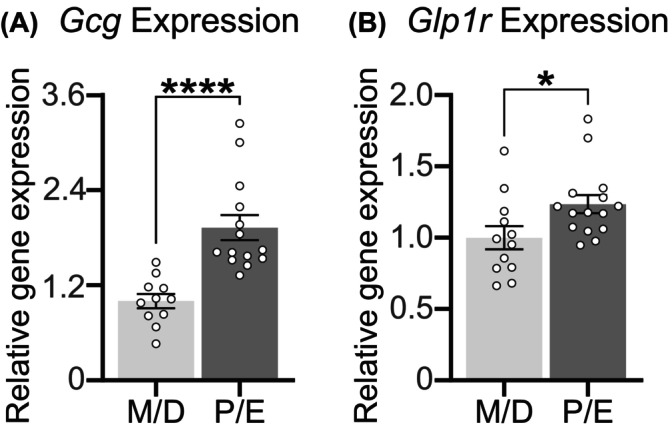
In female rats maintained on high‐fat diet, the nucleus tractus solitarius/area postrema contained greater (A) *Glucagon (Gcg)* expression (*p* < 0.0001) and (B) *Glucagon‐like receptor 1* (*Glp1r*) expression (*p =* 0.0286) during combined Proestrus/Estrus (P/E) compared to animals in Metestrus/Diestrus (M/D). *n* = 11–15/group, between‐subjects design. *p < 0.05; ****p < 0.0001.

To mimic chronic use of GLP‐1R‐based treatments, we next conducted a 24‐day, between‐subjects study using semaglutide. Figure 3 depicts the impact of consistent administration during P/E or M/D on the intake‐suppressive and body weight‐reducing effects of semaglutide. Relative to vehicle‐treated animals, semaglutide administered during either M/D or P/E significantly attenuated both cumulative (Figure 3A) and 4‐day binned noncumulative food intake (Figure 3B) (4, 8, 12, 16, 20, 24) time points (*p* < 0.05). Maximal cumulative food intake suppression occurred after 24 days; animals in M/D consumed 96.0 ± 9.12 g less and animals in P/E consumed 125.70 ± 8.94 g less (both *p* < 0.0001) than vehicle‐treated animals. Compared to injections during M/D, injections during P/E led to a greater suppression of cumulative food intake at days 8, 12, 16, 20, and 24 (all *p* < 0.05), culminating in the greatest difference in cumulative intake after 24 days when P/E injections reduced intake by 29.68 ± 8.41 g relative to semaglutide administered in M/D (*p* = 0.0016). In contrast, injections during P/E led to a greater suppression of noncumulative food intake within bins ending at days 8, 12, and 24 (all *p* < 0.05) with a trend for the day 20 bin (*p* = 0.0604), and no difference in noncumulative intake for the day 16 bin.

Semaglutide administered during either P/E or M/D produced a significant reduction in body weight percentage (Figure 3C) compared to vehicle‐treated rats at all time points except for day 8. At day 8, rats injected with semaglutide in P/E (*p* < 0.0001), but not M/D (*p* = 0.0573), showed a significant reduction. After 24 days, vehicle‐treated animals gained 4.95 ± 0.57%, M/D semaglutide‐treated animals lost 7.89 ± 1.06%, and P/E semaglutide‐treated animals lost 12.15 ± 1.55%. Semaglutide administered during P/E also produced a greater percentage of body weight loss compared to injections during M/D at days 8, 12, 16, 20, and 24 (all *p* < 0.05). By 24 days, semaglutide‐treated animals injected during P/E had lost 4.25 ± 1.38% more than semaglutide‐treated animals injected during M/D (*p* = 0.007).

### 3.4 *Glp1r* and Gcg expression across the estrous cycle in high‐fat diet‐maintained animals

We next evaluated whether G*lp1r and Gcg* expression levels would differ across the estrous cycle in the same HFD‐maintained female rats. Within the NTS/AP, *Gcg* (*p* < 0.0001) (Figure 4A) and *Glp1r* (*p =* 0.0286) (Figure 4B) were greater during P/E compared to M/D animals.

**FIGURE 5:**
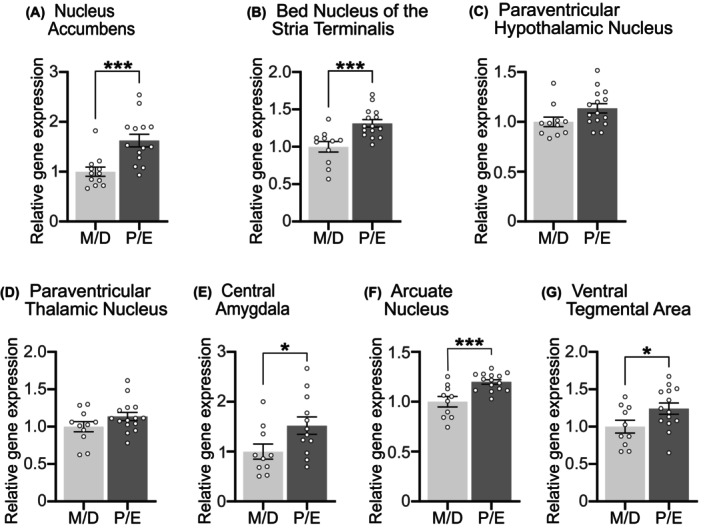
In female rats maintained on high‐fat diet, transcript expression of *Glucagon‐like receptor 1* (*Glp1r*) was greater during combined Proestrus/Estrus (P/E) compared to animals in Metestrus/Diestrus (M/D) in the (A) Nucleus accumbens, *p* = 0.007, (B) Bed nucleus of the stria terminalis, *p* = 0.009, (E) Central Amygdala, *p* = 0.0406, (F) Arcuate Nucleus, *p* = 0.006, and the (G) Ventral Tegmental Area, *p* = 0.0468, but not in the (C) Paraventricular Hypothalamic Nucleus, *p* = 0.0566, or the (D) Paraventricular Thalamic Nucleus, *p* = 0.1273. *n* = 10–15/group, between‐subjects design. *p < 0.05; **p < 0.01; ***p < 0.001.

Figure 5 depicts the impact of the estrous cycle on G*lp1r* expression in select nuclei within the cortex of the same animals. Compared to expression levels in M/D, animals in P/E showed increased *Glp1r* expression in the NAc (Figure 5A) and BNST (Figure 5B) (both *p* < 0.001), but not the PVN (Figure 5C) or PVT (Figure 5D). P/E *Glp1r* expression was greater in the CeA (Figure 5E), Arc (Figure 5F), and VTA (Figure 5G) (all *p* < 0.05).

## 4 DISCUSSION

Emerging data indicate most young adults prescribed GLP‐1RAs are women,^1^ underscoring a knowledge gap in preclinical GLP‐1 investigations which have inconsistently included females. Here, we aimed to address this by evaluating the impact of the estrous cycle on the intake‐ and body weight‐reducing effects of two different U.S FDA‐approved anti‐obesity medications. Importantly, our data do not show a direct role for changes in endogenous GLP‐1 in mediating the cyclical anorexigenic behaviour across the estrous cycle. We instead demonstrated that timing GLP‐1RA administration in P/E compared to M/D enhances the cumulative intake‐suppressive effects of liraglutide. While chronic semaglutide administration in P/E did not consistently produce a reduction in noncumulative intake in all 4‐day bins compared to M/D administration, it is encouraging that the reduction is sustained in the last time bin. Moreover, semaglutide injections in P/E produced a greater suppression of cumulative food intake compared to semaglutide administration in M/D, leading to significantly greater weight loss starting after 12 days of chronic semaglutide injections. We also highlighted a possible mechanism of action. Across multiple nuclei in lean and HFD‐maintained female rats, *Glp1r* expression increased in P/E. To date, this is the first study to show chronic semaglutide administration during P/E can increase weight loss relative to administration during M/D in rats.

Our food intake data are particularly notable because energy expenditure is increased in P/E, and partially accounts for the changes in body weight across the estrous cycle.^31^, ^52^ Our data points to enhanced GLP‐1RA suppression of food intake as a factor resulting in greater (whether additive or synergistic) weight loss in P/E. Factors regulating body weight in addition to food intake likely account for the lack of an interaction on body weight in our acute liraglutide experiment. While liraglutide administration during P/E interacted to potentiate food intake suppression, liraglutide and estrous cycle independently affected body weight. These separate effects together still produced the greatest body weight loss in liraglutide‐treated animals in P/E. Given the small interaction of liraglutide and P/E, it may be that chronic GLP‐1RA administration is required to observe potentiated weight loss. It is clear, however, that chronic semaglutide administration during P/E leads to greater weight loss compared to semaglutide administration in M/D. Regardless of mechanism (additive vs. synergistic) these findings, if translational, have meaningful implications for the relationship between estrous cycle and GLP‐1RAs.

Pharmacologic studies suggest that peaking estradiol in P modulates central mechanisms controlling food intake to produce anorexigenic effects that largely occur in E, when estradiol levels have returned to baseline.^31^ We speculate estradiol in P similarly provokes the *Glp1r* and *Gcg* increase that contributes to greater P/E GLP‐1RA effects. Indeed, we previously showed estradiol administration in males increased NTS/AP *Glp1r* and *Gcg* to levels resembling females in P/E.^40^ As circulating estradiol levels decrease following menopause, this highlights a need for GLP‐1 research in post‐menopausal females. Given the timing differences in estradiol peak to the onset of behavioural effects, combining P/E in our study limits our ability to determine which phase(s) of the estrous cycle potentiated GLP‐1RA's intake‐suppressive effects or increased *Glp1r* compared to M/D. Notably, the satiating ability of CCK is enhanced specifically in E (relative to D)^33^ and our previous data in lean animals showed increased NTS/AP *Glp1r* expression in E, but not P.^40^ It is thus likely that behavioural effects occurred predominantly in E. Yet, we also found increased *Gcg* NTS/AP expression in both P and E, which hypothetically may enhance GLP‐1RA sensitivity. Finally, variation in the speed/mechanism by which estradiol impacts gene expression may underscore the estrous‐cycle‐dependent effects of different satiation signals.

While acute liraglutide produced an expected increase in pica behaviour, we did not observe any estrous cycle‐dependent effect on kaolin intake. These data may suggest that relative to M/D, P/E administration can potentiate liraglutide's intake‐suppressive effects without provoking greater nausea/malaise. Nonetheless, women display more frequent nausea and vomiting compared to men, even at similar semaglutide exposure levels.^53^ Anecdotally, rats in P/E were more likely to dissemble kaolin into small pieces. While animals display increased physical activity during E,^54^ this confound likely increased error, and future studies may benefit from additional methods for tracking GLP‐1R‐induced malaise across the estrous cycle.

In both lean and HFD‐maintained animals, we showed that *Glp1r* expression during P/E increased in the NAc, BNST, CeA, Arc, and VTA. Moreover, HFD‐maintained female rats also increased NTS/AP *Gcg* and *Glp1r* during P/E, consistent with our previous data.^40^ When considered with our behavioural data, however, it is plausible that increased *Glp1r* in the aforementioned nuclei mediate the potentiated food intake‐suppressive effects of GLP‐1RAs in P/E relative to M/D. GLP‐1R‐expressing cells within the dorsal vagal complex, containing the NTS and AP, are necessary for the weight‐reducing effects of GLP‐1‐based obesity drugs.^9^, ^19^, ^47^ Moreover, fluorescently‐labelled semaglutide and liraglutide reach and provoke increased neuronal activity in the Arc, PVN, NTS/AP, and increase neuronal activity in the BNST and CeA.^55^, ^56^ Notably, exogenous estradiol impacts Glyceraldehyde‐3‐phosphate Dehydrogenase (*Gapdh*) expression in the hypothalamus^57^ and plausibly other brain regions. Although the impact of estradiol throughout the estrous cycle on *Gapdh* are unknown, our use of *Gapdh* as a housekeeping gene warrants cautious interpretation of our gene expression data.

The weight loss benefit of GLP‐1RAs appear to be most effective in patients who are younger and female.^58^ Physiological factors, including differences in body fat distribution and greater fat mass in women, nor differences in pharmacokinetics, entirely account for these sex differences in weight loss efficacy.^4^, ^59^ Yet, the root of these differences is otherwise unclear. Here, we observed estrous cycle‐dependent variation in *Glp1r* and *Gcg* that may explain some of these differences. Indeed, our previous research suggested that NTS/AP *Glp1r* and *Gcg* is increased during P/E relative to male levels.^40^ Considering this directionality, increased *Glp1r* and *Gcg* expression could enhance GLP‐1RA effects compared to males. Not only do our data highlight the necessity for sex‐specific considerations in obesity management, but also that pre‐ or postmenopausal status should be noted.

Data presented here suggest that GLP‐1RA administration during combined P and E can potentiate intake‐suppressive effects of GLP‐1RAs relative to administration in M/D, potentially leading to increased weight loss. If these findings are replicated in clinical literature, then prescribing physicians may need to consider menstrual cycle phase when treating women.

## CONFLICT OF INTEREST STATEMENT

The authors declare no conflicts of interest.

## Supporting information


Data S1.

## Data Availability

The data that support the findings of this study are available from the corresponding author upon reasonable request.
