# Supplementary material for: The estrous cycle moderates the food and body weight suppressive effects of glucagon‐like peptide‐1 receptor agonism
Source: Diabetes Obes Metab. 2025 Sep 29;28(1):221–30. doi: 10.1111/dom.70177 (PMC12673426; doi:10.1111/dom.70177)
Supplement: Supplementary file 1 — Data S1. [file DOM-28-221-s001.docx]

**MATERIALS AND METHODS**

Food, kaolin, and body weight measurements and calculations

We measured food and kaolin intake and body weight by determining cumulative change in the weight of the food hopper or the animal from time of injection to the indicated time point. To account for spillage of food or kaolin, we placed brown paper under each hanging wire cage to collect any crumbs and subtracted crumbs from weight change of the food or kaolin hopper, as we described previously^1,2^.

Brain Tissue Extraction

Following the 24-day chronic semaglutide experiment, all vehicle-treated animals recovered for 10 days. Semaglutide-treated animals recovered for a minimum of 4 weeks following day 24. Because semaglutide-treated animals received semaglutide injections on different dates within a four day period, the final injection for one rat could occur three days after the final injection for another rat. Moreover, to ensure adequate collection of brains from each phase, we extracted brains across 3 days, such that animals could be sacrificed either 1.4 weeks, 2.4 weeks and 1 day, or 3. 4 weeks and 2 days following day 24. Thus, semaglutide-treated animals recovered for a minimum of 4 weeks, and with a maximum of 4 weeks and 5 days. See Figure S2 for example timelines.

We sedated all rats using an intraperitoneal injection of an anesthetic cocktail comprised of ketamine (90 mg/kg; Covetrus North America), xylazine (2.8 mg/kg; Butler Animal Health), and acepromazine (0.72 mg/kg; Midwest Veterinary Supply) cocktail. Animals were sacrificed 3-4 hours before onset of the dark cycle. Feeding state was not manipulated.

We collected bilateral micropunches (0.75mm^3^) of the NTS/AP starting at Bregma -14.6mm to start of the 4th ventricle. We collected bilateral micropunches from the NAc (1mm^2^, Bregma 2.5 to 1.2mm), anterolateral BNST (1mm^2^, Bregma -0.2 to -0.9mm), CeA (1mm^2^, Bregma -1.75 to -2.75mm), and VTA (0.75mm^2^, Bregma -0.5.8 to -6.8mm). We collected unilateral mircopunches from the PVN (1mm^3^, Bregma -0.9 to -1.9mm), PVT (0.75mm^2^, Bregma -1.3 to -2.8mm), and Arc (0.75mm^2^, Bregma -2.3 to -3.6mm).

Quantitative Polymerase Chain Reaction (qPCR)

We processed brain tissue and performed qPCR as previously described^2^. Briefly, we purified total RNA from RNeasy Mini spin columns according to RNeasy Mini Kit (Qiagen). We determined concentration of RNA using a Qubit™ 4 fluorometer with a Qubit™ RNA HS Assay Kit (Thermo Fisher Scientific). We excluded samples with <5ng/µl of RNA. We synthesized complementary DNA as per Bio-Rad Reaction Setup for a Single cDNA Synthesis Reaction. We performed qPCR in duplicates using SsoAdvanced Universal SYBR® Green Supermix, (Bio-Rad) and QuantStudio 6 Flex Real-Time PCR system and software (Applied Biosystems). We used 2−ΔΔCT to analyze relative changes in gene expression, using housekeeping gene *Gapdh*. We normalized to mean ΔCt values derived from animals in M/D. Primer sequences are in Table S1.

**SUPPLEMENTAL TABLES**

**Table S1. qPCR Primers**

| mRNA name | Forward | Reverse |
| --- | --- | --- |
| *Gapdh* | CCG CAT CTT CTT GTG CAG TG | ACCAGCTTCCCATTCTCAGC |
| *Gcg* | AGTTCTCTTTCCAGGTTCACCAC | ACCGCCCTGAGATTACTTTTCTG |
| *Glp1r* | CCG GGT CAT CTG CAT CGT | AGTCTGCATTTGATGTCGGTCTT |

**Table S2. Statistical Analysis of Behavioral and qPCR Data**

| **Figure number** | **Factor name** | **F- or t-value** | ***p*-value** |
| --- | --- | --- | --- |
| 1A. Nucleus Accumbens Unpaired t-test | Phase (M/D, P/E) | *t*_35_=2.958 | 0.0055* |
| 1B. Bed Nucleus of the Stria Terminalis  Unpaired t-test | Phase (M/D, P/E) | *t*_35_=3.287 | 0.0023* |
| 1C. Paraventricular Hypothalamic Nucleus Unpaired t-test | Phase (M/D, P/E) | *t*_32_=0.9001 | 0.3748 |
| 1D. Paraventricular Thalamic Nucleus  Unpaired t-test | Phase (M/D, P/E) | *t*_35_=4.026 | 0.0003* |
| 1E. Central Amygdala  Unpaired t-test | Phase (M/D, P/E) | *t*_35_=3.417 | 0.0016* |
| 1F. Arcuate Nucleus of the Hypothalamus  Unpaired t-test | Phase (M/D, P/E) | *t*_34_=4.178 | 0.0002* |
| 1G. Ventral Tegmental Area  Unpaired t-test | Phase (M/D, P/E) | *t*_35_=2.079 | 0.045* |
| 2A. Cumulative Food Intake  Repeated-measures ANOVAs | **1 Hour**  Liraglutide (Veh, 50 μg/kg)  Phase (M/D, P/E)  Liraglutide x Phase  **3 Hours**  Liraglutide (Veh, 50 μg/kg)  Phase (M/D, P/E)  Liraglutide x Phase  **6 Hours**  Liraglutide (Veh, 50 μg/kg)  Phase (M/D, P/E)  Liraglutide x Phase  **24 Hours**  Liraglutide (Veh, 50 μg/kg)  Phase (M/D, P/E)  Liraglutide x Phase | *F*_1, 26_ = 0.4758  *F*_1, 26_ = 15.26  *F*_1, 26_ = 0.5956  *F*_1, 26_ = 28.87  *F*_1, 26_ = 40.68  *F*_1, 26_ = 1.187  *F*_1, 26_ = 67.23  *F*_1, 26_ = 54.51  *F*_1, 26_ = 3.361  *F*_1, 26_ = 374.0  *F*_1, 26_ = 90.47  *F*_1, 26_ = 10.68 | 0.4954  0.0006*  0.4472  <0.0001*  <0.0001*  0.2860  <0.0001*  <0.0001*  0.0782  <0.0001*  <0.0001*  0.003* |
| 2B. Percent Suppression of Cumulative Food intake Relative to Vehicle Controls  Paired t-tests | **24 Hours (2B)**  Phase (M/D, P/E) | *t*_26_=2.973 | 0.0063* |
| 2C. Percent Change in Body Weight  Repeated-measures ANOVA | Liraglutide (Veh, 50 μg/kg)  Phase (M/D, P/E)  Liraglutide x Phase | *F*_1, 26_ = 536.3  *F*_1, 26_ = 249.4  *F*_1, 26_ = 3.572 | <0.0001*  <0.0001*  0.0699 |
| 2D. Percent Body Weight Lost Relative to Vehicle Controls  Paired t-test | Phase (M/D, P/E) | *t*_26_=1.890 | 0.0699 |
| 2E. Kaolin Intake  Repeated-measures ANOVA | Liraglutide (Veh, 50 μg/kg)  Phase (M/D, P/E)  Liraglutide x Phase | *F*_1, 26_ = 77.55  *F*_1, 26_ = 0.00757  *F*_1, 26_ = 3.382 | <0.0001*  0.9313  0.0774 |
| 3A. Cumulative Food Intake  One-way ANOVAs | **4 Days**  Condition (Veh-MD+P/E, Semaglutide-M/D, Semaglutide-P/E)  **8 Days**  Condition (Veh-MD+P/E, Semaglutide-M/D, Semaglutide-P/E)  **12 Days**  Condition (Veh-MD+P/E, Semaglutide-M/D, Semaglutide-P/E)  **16 Days**  Condition (Veh-MD+P/E, Semaglutide-M/D, Semaglutide-P/E)  **20 Days**  Condition (Veh-MD+P/E, Semaglutide-M/D, Semaglutide-P/E)  **24 Days**  Condition (Veh-MD+P/E, Semaglutide-M/D, Semaglutide-P/E) | *F*_2, 26_ = 46.45  *F*_2, 26_ = 29.16  *F*_2, 26_ = 25.60  *F*_2, 26_ = 44.29  *F*_2, 26_ = 55.11  *F*_2, 26_ = 54.52 | <0.0001*  <0.0001*  <0.0001*  <0.0001*  <0.0001*  <0.0001* |
| 3B. Noncumulative Food Intake  Two-Way Mixed ANOVA | Time (4, 8, 12, 16, 20, 24 Days)  Condition (Veh-MD+P/E, Semaglutide-M/D, Semaglutide -P/E)  Time x Condition | *F*_5, 130_ = 16.29  *F*_2, 26_ = 54.93  *F*_10, 130_ = 3.475 | <0.0001*  <0.0001*  0.0004* |
| 3C. Percent Change in Body Weight  Two-Way Mixed ANOVA | Time (4, 8, 12, 16, 20, 24 Days)  Condition (Veh-MD+P/E, Semaglutide-M/D, Semaglutide -P/E)  Time x Condition | *F*_5, 130_ = 5.582  *F*_2, 26_ = 45.44  *F*_10, 130_ = 14.98 | 0.0001*  <0.0001*  <0.0001* |
| 4A. Nucleus Tractus Solitarius-*Gcg* Expression Unpaired t-test | Phase (M/D, P/E) | *t*_23_=4.737 | <0.0001* |
| 4B. Nucleus Tractus Solitarius-*Glp1r* Expression Unpaired t-test | Phase (M/D, P/E) | *t*_25_=2.324 | 0.0286* |
| 5A. Nucleus Accumbens Unpaired t-test | Phase (M/D, P/E) | *t*_24_=3.878 | 0.0007* |
| 5B. Bed Nucleus of the Stria Terminalis  Unpaired t-test | Phase (M/D, P/E) | *t*_24_=3.776 | 0.0009* |
| 5C. Paraventricular Nucleus of the Hypothalamus  Unpaired t-test | Phase (M/D, P/E) | *t*_24_=2.003 | 0.0566 |
| 5D. Paraventricular Nucleus of the Thalamus  Unpaired t-test | Phase (M/D, P/E) | *t*_24_=1.579 | 0.1273 |
| 5E. Central Amygdala  Unpaired t-test | Phase (M/D, P/E) | *t*_20_=2.189 | 0.0406* |
| 5F. Arcuate Nucleus of the Hypothalamus  Unpaired t-test | Phase (M/D, P/E) | *t*_23_=3.956 | 0.0006* |
| 5G. Ventral Tegmental Area  Unpaired t-test | Phase (M/D, P/E) | *t*_22_=2.107 | 0.0468* |

**SUPPLEMENTAL FIGURES**


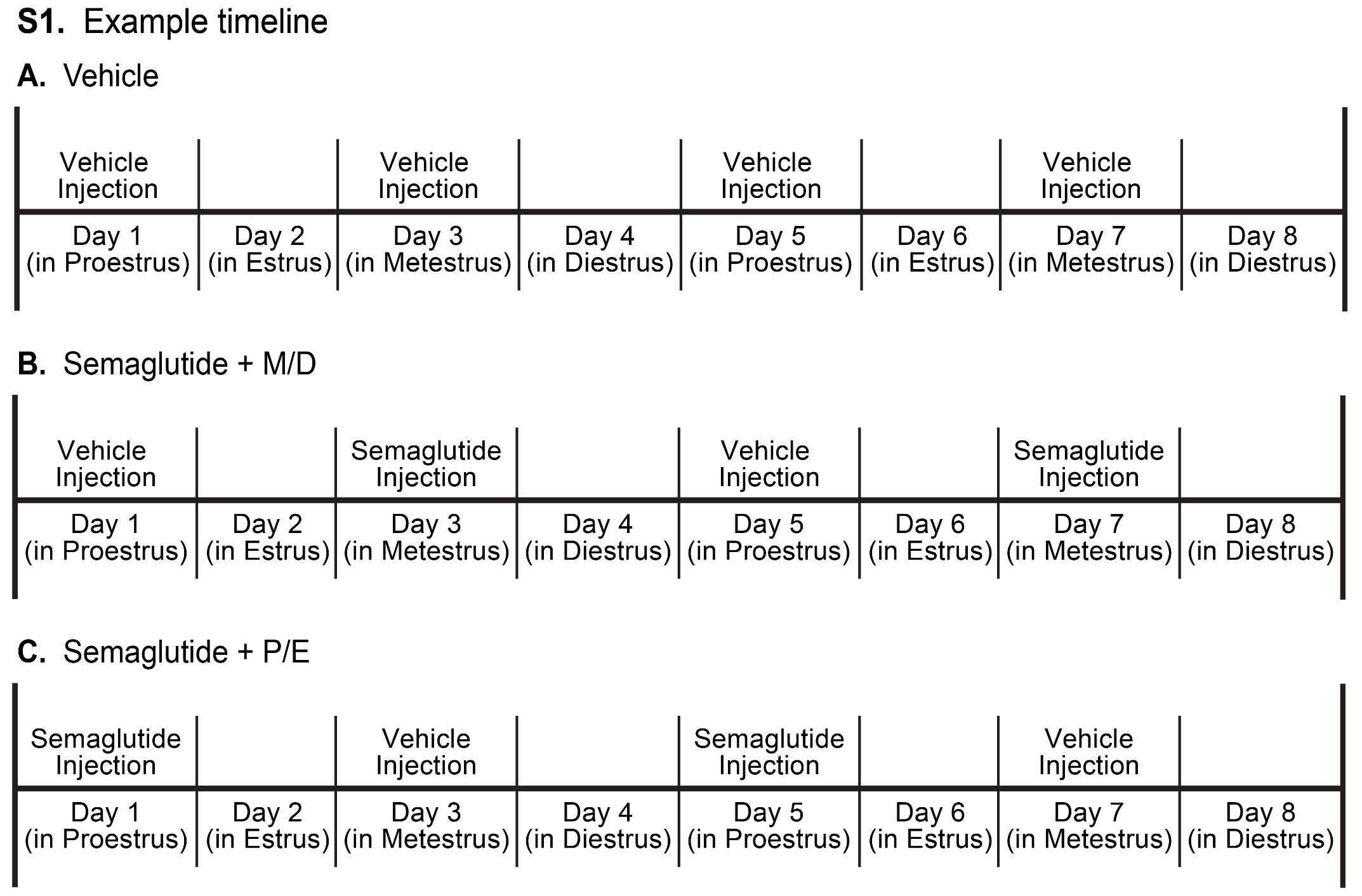


**Figure S1.** Example 8-day timeline for a rat in the chronic semaglutide experiment. (**A)** A rat assigned to the vehicle treatment condition would receive an injection during proestrus or estrus and metestrus or diestrus. In this example, the animal was injected with vehicle during proestrus, followed by another vehicle injection 48-hours later in metestrus. 48-hours later, the animal was injected again while in proestrus. (**B**) A rat assigned to receive semaglutide (20nmol/kg) during metestrus/diestrus (Vehicle + M/D) would receive a vehicle injection during proestrus or estrus and a semaglutide injection in metestrus or diestrus. In this example, the animal was injected with vehicle during proestrus, followed by a semaglutide injection 48-hours later in metestrus. While the next vehicle injection occurred 48-hours later in proestrus, exposure to semaglutide was separated by 96-hours on (Day 3 and Day 7). (**C**) A rat assigned to receive semaglutide (20nmol/kg) during proestrus/estrus (Vehicle + P/E) would receive a semaglutide injection during proestrus or estrus and a vehicle injection in metestrus or diestrus. In this example, the animal was injected with semaglutide during proestrus, followed by a vehicle injection 48-hours later in metestrus. Semaglutide next was injected on Day 5, 48-hours after the vehicle injection. Exposure to semaglutide was separated by 96-hours on (Day 1 and Day 5).


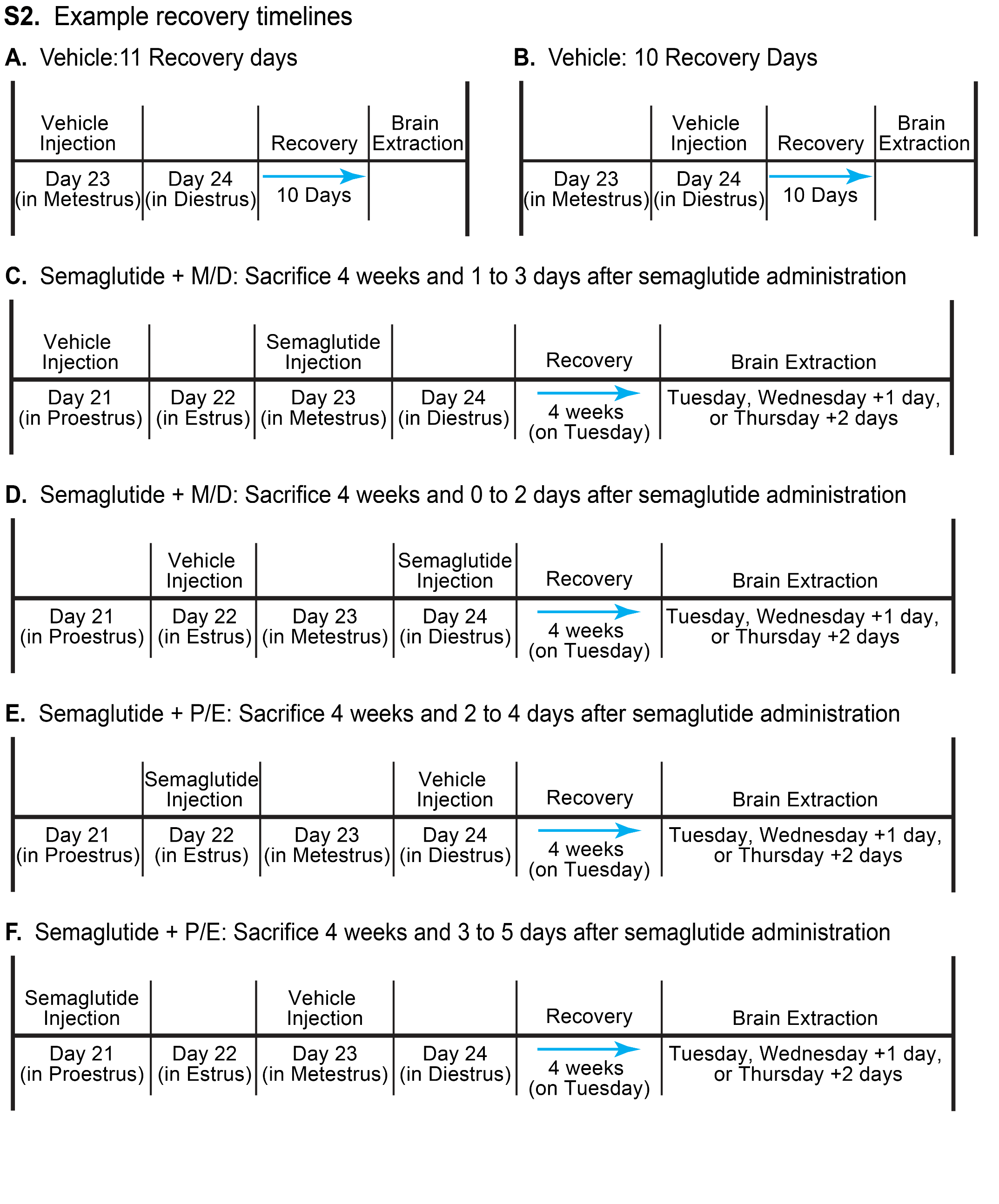


**Figure S2.** Example timeline for recovery and sacrifice following the chronic semaglutide experiment. (**A and B)** In the last two days of the experiment, a vehicle-treated rat would receive an injection during either day 23 or (**B**) day 24. All vehicle-treated animals recovered for 10 days after the experiment. Thus, an animal injected on day 23 was sacrificed 11 days after the final injection while an animal injected on day 24 was sacrificed 10 days after the final injection. (**C and D**) . A rat assigned to receive semaglutide (20nmol/kg) during metestrus/diestrus (Vehicle + M/D) would receive a semaglutide injection in metestrus (**C.** day 23 in this example) or diestrus (**D**.day 24 in this example). Animals were sacrificed beginning four weeks after day 24. Thus, an animal injected on day 23 would have at minimum, 4 weeks and 1 day between final semaglutide administration and brain extraction. Animals were sacrificed across three days, so that in the above example, and animal sacrificed four weeks after day 24 would be sacrificed on Tuesday, while an animal sacrificed on the last day (Thursday) in **C** would have not received semaglutide for 4 weeks and 3 days before sacrifice. Similarly, the animal in **D** injected on day 24 would have 4 weeks to 4 weeks and 2 days between semaglutide injection and sacrifice. (**E and F)** A rat assigned to receive semaglutide during proestrus/estrus (Vehicle + P/E) would receive a semaglutide injection during proestrus (**F**: day 21 in this example) or estrus (**E:** day 22 in this example). Animals were sacrificed beginning four weeks after day 24. Thus, an animal injected on day 22 would have at minimum, 4 weeks and 2 day between final semaglutide administration and brain extraction, while an animal sacrificed on the last day (Thursday) in **E** would have not received semaglutide for 4 weeks and 4 days before sacrifice. Similarly, the animal in **F** injected on day 21 would have 4 weeks and 3 days (sacrificed on Tuesday) to 4 weeks and 5 days (sacrificed on Thursday) between semaglutide injection and sacrifice.

**REFERENCES**

1. Kanoski SE, Rupprecht LE, Fortin SM, De Jonghe BC, Hayes MR. The role of nausea in food intake and body weight suppression by peripheral glp-1 receptor agonists, exendin-4 and liraglutide. Neuropharmacology 2012;62(5-6):1916-1927.

2. Applebey SV, Xiao AG, Harris EP, et al. Characterizing brainstem glucagon-like peptide-1 control of sensory-specific-satiety in male and female rats across the estrous cycle. *Biological Psychiatry* 2025.
